# Effect of Glass Fiber Reinforcement on Marginal Microleakage in Class II Composite Restorations: An In Vitro Pilot Study

**DOI:** 10.3390/dj12120410

**Published:** 2024-12-16

**Authors:** Csaba Dudás, Emánuel Kardos, Melinda Székely, Lea Ádám, Zsuzsanna Bardocz-Veres, Evelyn Szőllősi, Kinga Mária Jánosi, Bernadette Kerekes-Máthé

**Affiliations:** 1 Faculty of Dentistry, George Emil Palade University of Medicine, Pharmacy, Science and Technology of Târgu Mureș, 540139 Targu Mures, Romania; csaba.dudas@umfst.ro (C.D.);; 2 Department of Teeth and Dental Arches Morphology, George Emil Palade University of Medicine, Pharmacy, Science and Technology of Targu Mures, 540139 Targu Mures, Romania; bernadette.kerekes-mathe@umfst.ro; 3 Department of Oral Rehabilitation and Occlusology, George Emil Palade University of Medicine, Pharmacy, Science and Technology of Targu Mures, 540142 Targu Mures, Romania; zsuzsanna.bardocz-veres@umfst.ro; 4 Department of Fixed Prosthodontics, George Emil Palade University of Medicine, Pharmacy, Science and Technology of Targu Mures, 540142 Targu Mures, Romania; kinga.janosi@umfst.ro

**Keywords:** restorative approach, class II restoration, composite resin, fiberglass strip, dye penetration

## Abstract

**Background:** Polymerization shrinkage of composite resins affects the marginal closure of direct dental restorations. It is responsible for developing secondary caries and indirectly affects the survival rate of restorations. This study aims to investigate the null hypothesis, which states that there are no significant differences in the marginal microleakage of Class II restorations when examined in vitro using different dental adhesives, whether the restoration material used is a composite with glass fiber reinforcement or not. **Methods:** Class II cavities were prepared on both proximal surfaces of thirty-six extracted human molars. A single-component (Universal VivaPen) and a two-component (Futurabond DC) self-etch adhesive system were used for the restorations in the control group (Charisma Classic) and the experimental group (Charisma Classic with Interlig glass fiber strip). An oblique layering technique and a 40-s soft-start light-curing polymerization were used. After selective pre-isolation, the specimens were placed in a 0.2% methylene blue solution and incubated at 37 °C for 24 h. The teeth were sectioned in the mesiodistal direction, and two examiners examined and graded the extent of dye penetration. Statistical analysis was conducted using the Mann–Whitney U and chi-square tests (*p* < 0.05). **Results:** All the composite restorations reinforced with glass fiber showed significantly reduced dye infiltration compared to the control group (*p* < 0.05). A significant difference (*p* < 0.05) was also observed between the two adhesives. **Conclusions:** The null hypothesis was rejected. Glass fiber strips significantly reduced composite restoration microleakage regardless of the adhesive. The marginal fit of the restoration was also influenced by the adhesive system used.

## 1. Introduction

A quick search of the MEDLINE electronic database (via PubMed) used the following search string: (dental) AND (composite) AND ((microleakage) OR (microleakage)) OR (shrinkage); it yielded 39,308 results as of 12 August 2024. No language or study design restrictions were applied to the search. While the final number of relevant results would be reduced after screening, the fact remains that interest in this topic is growing every year. This becomes even more evident in 2022, which was an exceptional year with 4568 articles published. One possible reason for the popularity of this topic is that polymerization shrinkage cannot be eliminated entirely. It is the primary cause of marginal leakage and staining, secondary caries, cuspal deflection, postoperative sensitivity, and restoration fractures, all of which can ultimately lead to restoration failure. Clinically, achieving the best marginal adaptation to prevent secondary caries is pivotal. The best possible adaptation must be pursued for direct and indirect restorations [1]. Significant stress can develop at the tooth–restoration interface during polymerization shrinkage. The magnitude of this stress depends primarily on the characteristics of the materials and the light-curing process. However, the cavity configuration and the techniques used, such as layering or fiber strip application, are also critical factors [2].

Fiber-reinforced composites consist of at least two different components. The reinforcing component provides strength and stiffness, while the surrounding matrix provides workability. Dental restorations commonly use a resin matrix reinforced with glass, polyethylene, or carbon fibers. These fibers can be arranged in various configurations. The development of glass fiber-reinforced composite resins and the positive clinical results have led to their widespread use in many dental applications [3,4,5].

Glass fibers have been used primarily to construct post-root canal posts and reinforce composite restorations. The advantage of the latter application is that glass fiber strips increase the tensile strength of restorations, reduce the detrimental effects of the C-factor, moderate polymerization shrinkage and microleakage, and minimize stress concentrations by distributing forces over a larger area [6,7,8]. Some authors suggest that preheating bulk fillings and conventional composite resins to 50 °C reduces microleakage in the dentin margins of Class II cavities [8,9]. In a meta-analysis, Elkaffas et al. found sufficient scientific evidence that preheating can improve the hardness of resin composites [10].

Another area where the use of glass fibers is significant is the reinforcement of the base plate of removable dentures [11]. This approach is recommended for patients who have previously fractured their removable denture or are wearing an implant-retained overdenture [12,13]. Glass fibers are the most suitable for this purpose because they are translucent and can form an excellent chemical bond between the fiber and the polymer matrix with a silane. Glass fibers are also used as a temporary solution in direct fiber-reinforced composite fixed partial dentures (FRC-FPD) [14]. In a retrospective cohort study with a mean follow-up of 53 months on 100 dentures, Perrin et al. found that the cumulative survival rate was 93%, with 69% of the restorations still functioning without further treatment [15]. Under these circumstances, FRC-FPD can be an immediate, short- to medium-term solution for replacing one to two missing teeth with no or minimal tooth preparation.

In oral surgery, glass fibers have been used to restore teeth that have suffered various mechanical traumas and for complex cranial reconstructions. In addition, they can be helpful in the treatment of teeth with increased mobility in adults or children [16]. In pedodontics, glass fibers are versatile and suitable for use as space maintainers in both the posterior and anterior regions (FRC-FPD) and endodontic posts and cores [5]. Small-volume aesthetic retainers can also be fabricated after orthodontic treatment.

Therefore, the objective of the present in vitro study was to test our null hypothesis that there are no significant differences in the marginal microleakage of the Class II restorations in either those made using the studied composite resin with or without glass fiber reinforcement or those made using different dental adhesives. Dye penetration was evaluated to indicate marginal microleakage of the direct restorations.

## 2. Materials and Methods

A total of thirty-six caries-free human molars, extracted for orthodontic or periodontal indications, were selected and placed in a mixture of disinfectant solution and distilled water to eliminate pathogens and maintain the moisture of the tooth’s hard tissues. The sample size calculation was performed with an effect size of 0.8 and a significance level of 5% using G*Power 3.1.9.7 software (Düsseldorf, Germany). The study was approved by the Ethics Committee for Scientific Research of the G.E. Palade University of Medicine, Pharmacy, Science and Technology, Targu Mures, Romania (Approval No. 1895/19.10.2022). Written informed consent was obtained from the patients before tooth extraction.

Class II cavities were created by a single operator (E.K.) using a spherical diamond bur mounted in a dental turbine, which was replaced after use on nine teeth. The cavities were extended axially to the cementoenamel junction (CEJ) and were 3 mm in depth mesiodistally and 3 mm wide buccolingually in both proximal cavities (n = 72). The internal angles and edges of the preparation margin were rounded. To facilitate more accessible and more accurate filling, the teeth with prepared cavities were placed in a condensation silicone (Zetaplus, Zhermack S.p.A., Badia Polesine, Italy) mold. The cavities were then thoroughly rinsed with a water jet and dried with air.

The teeth were randomly selected into two main groups of eighteen specimens according to the adhesives used: Universal VivaPen (Ivoclar Vivadent AG, Schaan, Liechtenstein), a single-component self-etch adhesive, and Futurabond DC (VOCO GmbH, Cuxhaven, Germany), a two-component self-etch adhesive. Each group was randomly split into two subgroups in which composite resin fillings were placed with or without glass fiber reinforcement. One of the cavities in each tooth was randomly filled with composite restorative material alone (considered as the control groups, nos. 1 and 3, n = 36). In contrast, the other cavity was filled with composite resin combined with a glass fiber strip (considered as the experimental groups, nos. 2 and 4, n = 36). Randomization was performed using random number tables controlled with permuted blocks to ensure an equal number of specimens in each group, as shown in Table 1. Although both adhesives can be used with the self-etch technique, it is generally accepted that a better bond is achieved with an additional phosphoric etching [17,18,19]. Therefore, the enamel and dentin surfaces were etched with 37% phosphoric acid for 15 s. The acid was then rinsed off for 10 s, the surface was allowed to dry slightly, and the adhesive was applied and spread. Finally, the adhesives were photopolymerized for 10 s, as recommended by both manufacturers.

All the prepared cavities were filled with commercially available composite Charisma Classic (Kulzer GmbH, Hanau, Germany). A thin layer of composite was placed on the gingival wall of the cavity and exposed to light emitted from a cordless LED curing light (NOBLESSE, Max Dental Co., Bucheon, Republic of Korea) in the soft-start curing mode for 40 s. In the control group cavities, the composite was placed using the oblique layering technique until the occlusal surface was reached. The layers were photopolymerized, as described above. In the cavities of the experimental group, after a thin layer of composite was applied to the gingival wall, a 2.5 mm pre-cut glass fiber strip (0.2 mm thick and 2 mm wide) (Interlig (IL), Angelus Indústria de Produtos Odontológicos S/A, Londrina, Brazil) was inserted before polymerization. The additional composite layers were then placed in the cavity, in a similar manner to the abovementioned technique.

Two coats of transparent nail polish were applied to the teeth after the fillings were finished, avoiding a 2 mm area around the gingival margin of the restoration. Self-curing acrylic resin was used to seal the apex of every root. The samples were then put into plastic test tubes filled with a 0.2% methylene blue solution. The test tubes were stored in an incubator (Cultura, Ivoclar Vivadent AG, Schaan, Liechtenstein) at 37 °C for 24 h to simulate the oral environment.

After removal from the dye solution, the specimens were thoroughly washed and dried. Translucent, self-curing acrylic resin (Duracryl Plus, Spofa Dental, A Kerr Company, Nymburk, Czech Republic) was placed in square molds, and the teeth were inserted before the material was cured. On the cured acrylic blocks thus formed, we recorded which side of the tooth received the glass fiber strip for filling and which adhesive was used for the restoration. Each tooth was then axially sectioned mesiodistally in the middle of the restoration using a precision cutter (Micracut 151, Metkon Instruments Inc., Bursa, Turkey). 

**Figure 1:**
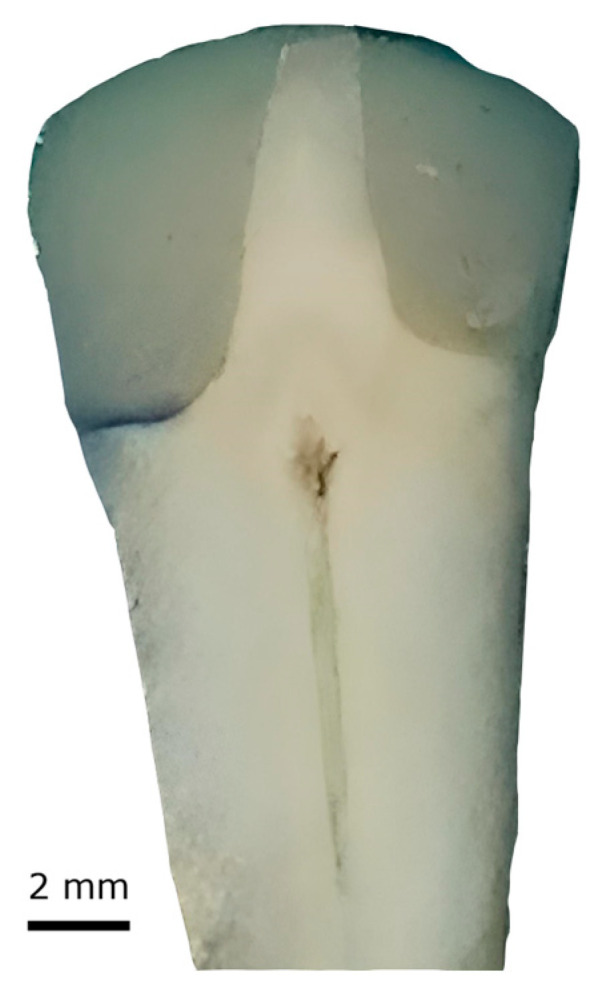
Example of dye penetration: Note that one cavity was filled with composite alone (left side, where dye penetration is visible), while the other was filled with composite resin combined with a glass fiber strip (right side with no dye penetration).

Standardized photographs were taken with a DSLR camera (Nikon D3100, Nikon Corporation, Tokyo, Japan) using a 90 mm macro lens (Tamron SP AF-S 90 mm, f/2.8, Tamron Co., Saitama, Japan), a cable shutter (Nikon MC-DC2), and a photo tent with a scattered light source. The degree of methylene blue penetration was examined using Image Pro Insight 8.0 (Media Cybernetics, Rockville, MD, USA) software. According to the degree of penetration, a scale with six grades was used to evaluate the marginal adaptation of the dental restorations [20]. The grades were defined as follows: (0) no visible dye infiltration at the gingival margin of the restoration; (1) dye infiltration involving half of the gingival wall; (2) dye infiltration involving the entire gingival wall; (3) dye infiltration involving ⅓ of the pulp wall; (4) dye infiltration involving ⅔ of the pulp wall; and (5) dye infiltration involving the entire pulp wall. Two examiners (E.K. and C.D.) independently assessed the extent of dye penetration for each selected tooth section. A third examiner (B.K.M.) reviewed the section and provided the final determination if a disagreement arose (Figure 1).

All the data obtained were entered into a spreadsheet, and statistical analysis was performed using IBM SPSS 29 software (SPSS Inc., Chicago, IL, USA). The Shapiro–Wilk test was used to test the data for normal distribution. The non-parametric Mann–Whitney U test and chi-square tests were used for inferential statistics. The significance level was set at *p* < 0.05.

## 3. Results

Dye penetration assessments were made on both sides of the specimens. The penetration depth of the methylene blue dye solution was scored according to the scale presented above. Table 2 shows the dye penetration data obtained in the different subgroups: grades, mean values, and standard deviation (SD).

**Figure 2:**
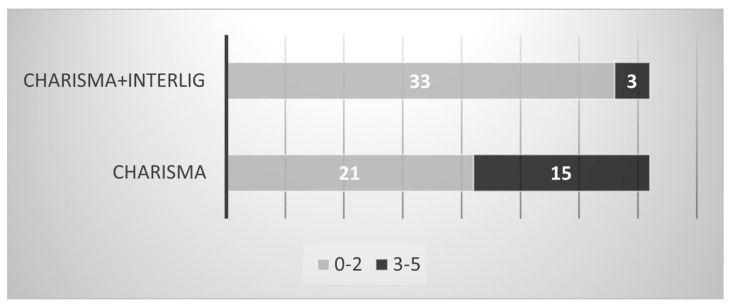
Control and experimental groups according to the degree of dye penetration. Light grey shows grades between 0 and 2; dark grey indicates grades between 3 and 5.

The chi-squared test revealed a statistically significant difference in the degree of dye penetration between the control (Charisma) and experimental (Charisma + IL) groups (*p* = 0.001). Figure 2 shows the number of values with no dye penetration or penetration extending only to the gingival wall (grades 0–2) and sections where the dye reached the pulpal wall or extended on the pulpal wall (grades 3–5). In addition, the Mann–Whitney U test showed a significant difference in microleakage grades between the control and experimental groups (*p* = 0.033).

The efficacy of the two different adhesives was also compared. Adhese Universal VivaPen showed significantly better results in both the control and experimental groups than Futurabond DC (*p* < 0.05). When the results were grouped according to the restoration technique used (Charisma or Charisma + IL) and the adhesive systems used, the Kruskal–Wallis test revealed statistically significant differences between the groups studied (*p* = 0.005).

## 4. Discussion

This in vitro study chose a micro-hybrid composite for cavity restoration. General dentists commonly use this type of material; however, its prevalence rate varies in different cross-sectional studies [21,22]. Its advantages include universal applicability in anterior and posterior areas, polishability, and affordability [23]. It is widely accepted that nanofilled composites represent the current state of the art. The nanoparticles in these materials provide improved microhardness and shade stability, although a decrease in these properties over time has been documented [24,25].

Fiber placed on the gingival wall of Class II cavities can improve restoration quality. Firstly, the fibers replace a part of the composite volume, which reduces polymerization shrinkage. Secondly, they help the first layer of composite resist detachment from the cavity walls during photopolymerization. Glass fibers, which are translucent, provide a more effective reinforcing effect than polyethylene fibers due to better adhesion between the glass fiber and the resin matrix [26,27].

Several methods have been used to detect microleakage, including air pressure, artificial caries, radioactive isotopes, scanning electron microscopy, and microcomputed tomography [28,29,30]. Even so, dye penetration remains the most commonly used method for in vitro examination of microleakage [27]. Although this test has certain limitations, such as the subjectivity of the readings, it is easy to perform with accurate measurements. In addition, it is inexpensive, provides excellent contrast to the dental material, and has no radiation risk. Basic fuchsine, methylene blue, and silver nitrate are commonly used as dyes [8]. We used a 0.2% methylene blue dye solution in our study for these reasons.

The layering technique affects marginal microleakage in Class II composite restorations. Several authors prefer the oblique layering technique over other methods because it reduces the C-factor. Studies have shown that microleakage was decreased significantly in groups where the composite resin was applied layer by layer compared to groups where the bulk-fill technique was used [31,32].

The soft-start polymerization technique is another method of reducing polymerization shrinkage [33]. During the first 10 s of illumination, low light intensity is used to delay the formation of cross-links between the polymers, which slows the curing of the resin. This allows the resin to remain in the gel phase longer, reducing shrinkage stress [34]. In addition, applying a surface sealant after finishing and polishing a direct composite restoration appears to seal marginal microleakage on the buccal and oral surfaces. However, it does not solve the marginal microleakage of proximal cavities [35].

Class II cavities were included in this study because their restoration with composites has long been debated among authors. The restoration of these cavities is a technique-sensitive process that presents many challenges to the practitioner, including time, application of the layering technique, limited direct vision, and difficulty in moisture control. Because marginal microleakage is lower at the enamel margins, greater precision and attention are required for cavities at the cementoenamel junction [36]. In addition, in practice, a significant amount of tooth material must often be removed. For these reasons, we extended the gingival margin of the cavity to the CEJ in our specimens.

It can be observed that the mean dye penetration values of the Charisma Classic direct composite restorations are higher than those of the fillings in the experimental group, in which a glass fiber strip was used in addition to the resin. The polymeric matrix composition of Charisma included Bis-GMA (bisphenol a diglycidyl ether dimethacrylate). It was characterized by the presence of barium aluminum fluoride glass (micro-glass) filler particles ranging from 0.7 to 2 μm. It is suggested that TCD-based nanohybrid resins have higher mechanical properties compared to Bis-GMA-based materials [33]. In this study, the cavities of all the groups were provided with a micro-hybrid composite resin, which is unsuitable for the bulk-fill technique. Based on the available literature, no statistically significant differences in the polymerization shrinkage of conventional and bulk-fill composites were observed [37,38,39,40]. Some authors have found that tested bulk-fill composites have similar marginal microleakage compared to conventional composites [41,42].

The manufacturers reported that both adhesives used in this study were compatible with all etching techniques, including self-etch. Adhese Universal VivaPen is a light-cure adhesive, while Futurabond DC is a dual-cure self-etch adhesive. According to Lührs et al., enamel’s additional phosphoric acid etching should be considered when using self-etch adhesives [17]. Other authors also confirmed that supplementary conditioning of dental surfaces significantly increased the shear bond strength of the examined self-etch adhesives [18,19]. Some studies have reported that Futurabond DC has significantly lower shear bond strength than other light-cured adhesives [43,44]. However, evidence also supports the contrary [45,46,47]. One thing is for sure: a randomized clinical trial with a one-year follow-up found that non-carious cervical lesions restored with the above adhesive had retention and success rates similar to those of other adhesives studied. However, the authors noted that the clinical results were inferior when Futurabond DC was applied using the self-etch method [48].

This study investigated the effect of glass fiber strips on the microleakage of composite restorations. The dye penetration was significantly reduced in the composite restorations where glass fiber strips were used. On the one hand, the fiber strips replace a part of the composite restoration near the gingival margins, thus resulting in less volumetric polymerization shrinkage. On the other hand, fibers may strengthen the composite margins, resisting deformation and resisting movement toward the light source from the margins [49]. Dhingra et al. showed that fiber-containing composites perform slightly better in terms of less microleakage than fiber-free composites. However, the difference was not statistically significant [50]. 

Noaman et al. used a different method to study polymerization shrinkage. Cuspal deflection, which is a biomechanical phenomenon, involves the displacement of cusps due to the interaction between the polymerization shrinkage stress of the composite and the tooth walls. By measuring cuspal deflection, they reported that using fiber inserts in resin composite restorations significantly reduced the microleakage values [51].

In a study, Özüdoğru et al. evaluated the effects of different methods of polyethylene fiber placement on the fracture resistance and microleakage of MOD cavities in molars [52]. They found that fractures occurring after resistance tests were more restorable in specimens with polyethylene fiber inserts compared to the control group. In addition, the microleakage values of restorations containing polyethylene fibers were significantly lower. Even so, not all authors agree on the efficacy of polyethylene fibers. In a 6-month aging study, samples were subjected to 3000 thermal cycles. Sharafeddin et al. concluded that polyethylene fiber inserts did not affect microleakage in Class II resin composite restorations with gingival margins below the CEJ [53]. 

A limitation of the present study might be the relatively small number of samples, which may affect the generalizability and robustness of the conclusions. Additionally, using a single composite material and two adhesives restricts the applicability of the findings to other commercially available composites and adhesives that may have different properties. The study design was an in vitro laboratory experiment, which cannot fully replicate the complex environment of the oral cavity. Factors such as microscopic cracks at the dentin and enamel levels and changes in humidity may affect the findings. Therefore, there may be differences between the findings of laboratory experiments and clinical observations. We plan to extend the research to include more types of composites and glass fiber strips, using advanced technologies (e.g., micro-CT scanning) to provide greater precision and improved visualization of marginal integrity.

According to Wallace et al., the quality of evidence is partially determined by the hierarchy of study designs [54]. Clinical observational research, represented by cross-sectional or cohort studies, occupies higher levels on the evidence pyramid than basic research under experimental conditions. Randomized controlled trials and systematic reviews and meta-analyses represent the highest quality of evidence [54]. More studies are needed to investigate the microleakage or survival rates of restorations made from the assessed materials. In a systematic review and meta-analysis, Escobar et al. examined data from 24 in vitro studies from the last 10 years concerning fracture resistance [55]. Based on the results, the authors demonstrated that the fracture resistance of fiber-reinforced (glass and polyethylene) composite restorations was higher than that of the controls: the composite restorations without fiber reinforcement and the unrestored cavity preparations.

## 5. Conclusions

Based on the results, the null hypothesis formulated was rejected. Within the limitations of this in vitro study, it can be concluded that the use of glass fiber strips significantly reduced microleakage in resin composite restorations of Class II cavities, regardless of the adhesive used. The teeth treated with Adhese Universal VivaPen adhesive showed significantly lower levels of dye penetration, regardless of whether a glass fiber strip was used. Therefore, extrapolating from the results obtained under experimental conditions, the clinical recommendation of this study would be that glass fiber strips could reduce the microleakage of composite restorations.

## Figures and Tables

**Table 1:** The control and experimental groups of the study (n = 72).

Group	Type	Adhesive Used	Restoration Method
1	control	Universal VivaPen	Charisma Classic
2	experimental	Universal VivaPen	Charisma Classic + glass fiber strip
3	control	Futurabond DC	Charisma Classic
4	experimental	Futurabond DC	Charisma Classic + glass fiber strip

**Table 2:** Summary of dye infiltration data in the four study groups. The number of grades obtained in each group and the mean values with standard deviations (SD) are shown.

Group	Grade 0	Grade 1	Grade 2	Grade 3	Grade 4	Grade 5	Mean	SD
1	4	7	3	4	-	-	1.39	1.09
2	6	6	4	2	-	-	1.11	1.02
3	2	2	3	8	1	2	2.56	1.42
4	1	8	8	1	-	-	1.50	0.70

## Data Availability

The data presented in this study are available on request from the corresponding author.
